# Management Of Recent Elbow Dislocations: Functional Treatment Versus Immobilization; A Prospective Study About 60 Cases

**DOI:** 10.2174/1874325001711010452

**Published:** 2017-05-30

**Authors:** Ndeye Fatou Coulibaly, Niane Mouhamadou Moustapha, Hamadi Hadji Djoumoi, Sarr Lamine, Gueye Alioune Badara, Sane André Daniel

**Affiliations:** 1 Department of Orthopedics Traumatology CHU le DANTEC Dakar, Senegal; 2 Department of Orthopedics Traumatology Regional Hospital of Thies, Senegal

**Keywords:** Elbow, Dislocation, Reduction, Immobilization, Functional treatment, Rehabilitation

## Abstract

**Objective:**

To determine our therapeutic posture trough a comparison of functional treatment results versus immobilization in two different periods.

**Introduction:**

For years, the treatment of recent elbow dislocations consisted of reduction and immobilization during 21 days. Given the frequency of stiffness other methods have been tried out.

**Method:**

A prospective study was carried out from January 2010 to December 2014. Sixty patients averaging 28.3 years of age underwent elbow dislocation reduction. They were categorized into three separate groups. Patients in the first group had their elbow immobilized for 21 days whereas Group 2 patients were immobilized for 10 days. Group 3 patients were applied a functional treatment followed by a functional rehabilitation. Patients were evaluated according to the Mayo Clinic Elbow Performance Index and the results analyzed with statistical software (SPSS, version 18).

**Results:**

During the first month, the functional results of the patients were excellent and good in 19%, 94.7% and 90% respectively for Groups 1, 2 and 3. The pain was intense (10 on the visual analogue scale) in group 3 associated with swelling. At day 90, the results of the patients in Groups 2 and 3 were excellent in 100% of the cases versus 90% for Group 1. At 6 months, all the results were the same. We have not noted any instability, or recurrence or periarticular ossification in our patients.

**Conclusion:**

The treatment of stable elbow dislocations remains orthopedic. The risk of instability and pain motivates a short 10-day immobilization period followed by early mobilization.

## INTRODUCTION

Dislocation represents 10% of elbow injuries [1], thus, ranking second in major dislocation cases after that of shoulder [2, 3]. It is usually observed with adolescents and young adults [1]. It generally occurs during sports injuries by an indirect mechanism [4]. Their diagnosis is quite easy after a physical exam. In benign appearance, they are sometimes accompanied by bone, nerve and /or vascular lesions. In such cases, management issues are delicate and the prognosis reserved [1]. Orthopedic treatment through reduction and contention is left most of the time to less experienced hands [5]. Elbow contention may be explained as a way of maintaining reduction through stability provision and allowance for the healing of ligament within the first 21 post traumatic days. The brachio-forearm plaster, which is called strict immobilization, keeping the elbow flexed to 90 degrees during this period, is not without consequences. Stiffness is the main complication, requiring long sessions of rehabilitation, reaching even arthrolysis to allow more or less complete functional recovery.

This raises a set of problems linked to immobilization after elbow dislocation reduction. Some schools offer to let elbow free of any contention after a steady reduction or, at most, to set up a scarf [1, 6]; this method is also called functional treatment. Others limit the contention to 15 days [7-9]. But whatever the type of immobilization, rehabilitation is an important step in their management.

The aim of our study was to determine a therapeutic stand by comparing the results of functional treatment versus immobilization allowing two distinct periods after reduction.

## MATERIALS AND METHODS

A prospective study covering a four-year period from January 1^st^, 2010 to December 30^th^, 2014 dealt with patients received in emergency for recent traumatic elbow dislocation. Inclusion criteria ranged from isolated dislocations to dislocations associated with a fractured radial head type 1 of Mason to fracture of the coronoid process type 1 of Morrey followed beyond 12 months. In total, 60 patients were selected.

Average age was 28.3 years (ranging from 15 to 64 years) with a standard deviation of 11.7. There were 51 men and 9 women with a sex ratio of 5.78. Sports injuries were a leading cause with 22 cases (36.7%), followed by household accidents (17 cases: 28.3%), highway accidents (13 cases: 21.7%), road traffic accidents (5 cases: 8.3%), accidents in the workplace (2 cases: 3.3%) and 1 fight (1.7%). The mechanism was indirect through falling and landing on the hand palm in 86.7% of cases while other imprecise cases stood at 13.3%.

Admission time averaged four hours after the trauma (range: 1 to 9 hours). This was an inaugural episode in all cases. Lesion in 36 cases (60%) concerned non-dominant upper extremity (27% right elbow and 73% left elbow). All injuries were closed.

Radiographically, three types of dislocations were found: posterolateral dislocation in 47 cases (78%), followed by genuine posterior dislocation (9 cases: 15%) and posteromedial dislocation (4 cases: 7%). Isolated dislocations were spotted in 48 cases (80%). Other patients showed associated injuries such as fractured radial head type 1 of Mason in 9 cases (15%) and injuries related to coronoid process type 1 of Morrey in 3 cases (5%).

Treatment consisted in reduction followed by contention.

We randomly divided patients into three groups according to the contention mode (Table **1**). Patients with a fractured radial head or a fractured coronoid process, were randomized in the group of patients immobilized for 10 or 21 days. A rehabilitation and medical treatment protocol based on analgesic and anti-inflammatory was carried out in all cases. Indomethacin was not prescribed in any of the cases. Massages or any possible application on the elbow were proscribed especially in patients under scarf. Patients were revisited in relationship to relevant immobilization type respectively on the following days: 7, 14, 30 and 90. In addition, a re-evaluation took place after six months and one year thereafter and on demand.

Patients were evaluated with the Mayo Clinic Elbow Performance Index [10]. Depending on final score, our results were classified as excellent, good, average or bad.

### Statistical Analysis

Results were analyzed with statistical software (SPSS, version #18). A paired T-test and logistical and linear regression analysis using the Pearson's Chi-squared test was also performed. The statistical test was considered significant if the p value was below 0.05.

## RESULTS

Dislocations were reduced within 15 minutes on average (range 10 to 75 minutes) after confirmation by radiography. The reduction was performed without anesthesia in 56 patients (93,3%), under sedative (diazepam) amongst 2 other patients (3.3%) while an additional 2 remaining patients (3.3%) were under general anesthesia. Protocol was carried out through external manipulations using the Fontaine technique [5]. Further, a testing was carried out in the flexion/extension arc on all patients whose reduction was performed without sedation (93,3%). Additional varus/valgus testing was applied on patients (6.6%) who had reduction under general anesthesia or sedation. These patients’ elbow was stable.

**Fig. (1):**
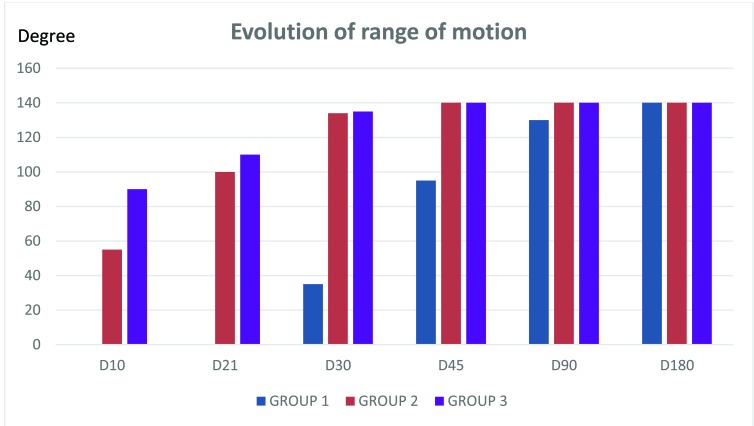
Evolution of range of motion.

All patients in Group 1 (G1) went on average through 12 sessions (ranging from 10 to 15) of functional rehabilitation after the removal of the plaster with the assistance of a physiotherapist. A physiotherapist assisted four patients in Group 2 (G2) through an average of five sessions (range: 3 and 7). A self-rehabilitation started on day 1 was adequate for Group 3 (G3) patients. Evolutionarily, from day 30, global functional results obtained during short 10-day immobilizations (G2) and early mobilizations starting from day one (G3) were met (Fig. **1**). Judging from the Mayo Clinic Elbow Performance criteria, they were excellent and good in 19%, 94.7%, and 90% respectively for group 1, 2 and 3 (Table **2**).

The results were statistically significant between G1 / G2 (p = 0.0001) and G1 / G3 (p = 0.0001). However, there were no statistically significant differences between G2 and G3 (p = 0.579).

**Fig. (2):**
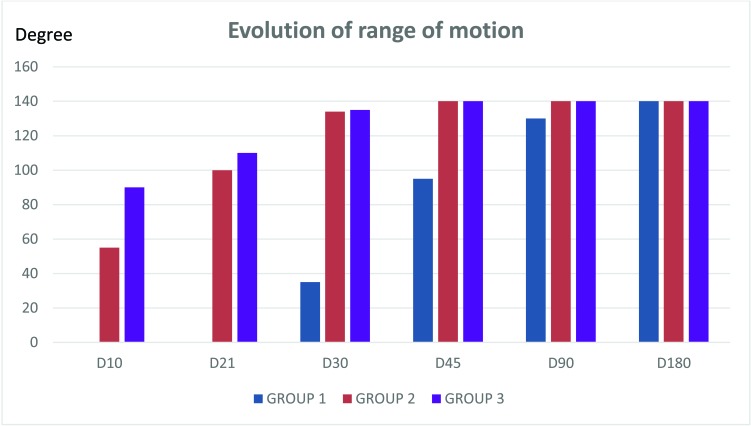
Evolution of pain.

During the first month, pain was intense; 10 on the Visual Analog Scale (VAS) associated with swelling in group 3. Pain evolution for each group is summarized in (Fig. **2**).

At day 90, the functional results of Group 1 almost equaled those of the other two groups. At 6 months, functional results of the 3 groups were the same (Fig. **1**). According to the Mayo Clinic elbow performance criteria results as of day 90 for patients in Groups 2 and 3 were excellent in 100% of cases versus 90% for Group 1 (Table **3**). No statistically significant differences were found between G1 / G2 (p = 0,157) and G1 / G3 (p = 0,168).

Regarding bone associated lesions, we found no differences between patients in the same group who had isolated dislocations on Day 30 and Day 90. No other complications (secondary displacement, malunion, periarticular ossification or osteoarthritis) were observed.

With a retrospect of 2 years 9 months on average (range: 1 to 4 years), we observed no recurrence of elbow dislocation or instability.

## DISCUSSION

It appears from this study that functional treatment (Group 3) and short 10-day immobilization period (Group 2) are similar in terms of elbow range of motion recovery. For Group 1 (21-day immobilization), the amplitude of patient’s mobility progressed slowly. Only after three months of physical therapy does it reach a maximum level of active flexion-extension.

This pathology care usually requires emergent treatment. However, in our case, it took a much longer period of treatment (four hours on average). A majority of our patients came from suburban areas where a large population of young people with sports activities and varied leisure were concentrated. They usually came with their own means of transport. Structures of emergency traumas exist in these areas but traumatology specialists were still in dire need. Treatment of isolated dislocations was always nearly orthopedic by external maneuvers. Several techniques were used, all responding to the same principle. Reduction under general anesthesia (RUGA) was preferred or whenever such protocol would be out of reach, sedation was the ideal solution. For RUGA allowed gentle gestures and a relaxed testing of stability on patients. However, in our context, the unavailability of surgical blocks and anesthetists made this stage difficult for us with only 6.6% of dislocations reduced respectively under general anesthesia (3.3%) and sedation (3.3%).

We applied the Fontaine reduction [5] for all of our patients not losing the sight of O'Driscoll *et al.* [11] recommendation to perform it on supine. This method may have provided greater stability during the dislocation treatment [11-14]. Surgical treatment is reserved to incoercible, inveterate dislocations or in cases of neurovascular lesions [16, 17]. Josefsson *et al.* [15] presented one of a few series when isolated dislocations were operated on to observe the extent of capsular ligament injuries in order to repair them. The proportion of post-operative elbow stiffness is higher in this series amongst these patients compared to patients treated using orthopedic procedures in the same series and in others [7, 18, 19].

Reduction was the overriding urgency in their treatment. Maintaining this reduction was the second step, the third one being to allow a capsular ligament healing in order to get a functional elbow during the various activities of daily life.

If the reduction technique is accepted by most schools of thoughts, the post reductional care still raises controversy. For a long time, immobilization by brachio-forearm plaster was systematic. It is analgesic and a stabilizer. The increased incidence of stiffness after removal of the gutter has questioned again this systematic immobilization process [15, 20]. It does not fight against capsular ligament retraction and, if anything, it may maximize the extant of stiffness with healing occuring when elbow is bent at 90° [11]. This questioning made it possible to introduce another concept in the management of reduced and stable dislocations: functional treatment or early mobilization. Consequently, on the one hand, immobilization supporters insist on its analgesic qualities, allowing capsular ligament healing and short as well as long-term stability [9, 16, 17, 21]. On the other hand, functional treatment supporters hold immobilization as a culprit for stiffness especially if prolonged [7, 8, 18, 19]. As a result of these competing views, works comparing immobilization and early mobilization were carried out [7, 14, 19]. Scholarly research comparing two groups of patients, one of which had an average immobilization of 21 days and the other an early mobilization, have displayed excellent results in the group of patients treated using functional treatment [7, 8, 18, 19, 22].

Other scholars have compared three groups of patients immobilized respectively during 14, 21 and 30 days after elbow reduction. Results were better in instances when immobilization did not exceed 14 days [9, 17, 22]. Straddling these two views, we decided to compare three patient groups, one of which had a functional treatment and the other two, immobilization for different durations. Thus, we studied an additional group compared with other studies carried out for this purpose (with or without immobilization).

The following results were obtained: in Group 3 where mobilization is done from the first day, inflammation is important in the form of swelling and intense pain. Similarly, our observation of patients led us to believe that stability provided by means of a scarf is elusive contrarily to results obtained amongst Group 2 patients. In this particular group, the 10-day period of short immobilization offers a comfort to the elbow, moderately reduces pain and inflammation and even allows an early start of rehabilitation before capsular ligament healing. However, there is no statistically significant difference in elbow motion amplitude recovery in these 2 groups. Further, we observed that from Day 30, mobility curves meet and remain inseparable with an optimal level of active flexion and extension.

On the other hand, amongst Group 1 patients, the amplitude of patients’ mobility was progressing slowly due to stiffness that occurred after immobilization. During a month period, we noticed a large percentage of poor results in this group in ten cases (16.7%). Only after three months of rehabilitation did they reach a maximum level of active flexion-extension.

The combination of a dislocation and a fracture of the coronoid process by Morrey type 1 or radial head by Masson type 1 had no influence on the evolution and functional recovery. Patients who presented these lesions were all treated by reduction and immobilization to get a stable fracture and avoiding any travel during that period of time. There was no difference in the recovery of mobility amplitudes compared to simple dislocation among those who received the same treatment protocol.

It appears from this study that the functional treatment (Group 3) and the short immobilization period of 10 days (Group 2) are equal in terms of recovery of elbow range of motion.

Given the best comfort provided by indolence, an inflammation decrease at the cost of short immobilization displayed among Group 2 patients may offer in our view far better results whether subjectively or objectively. These results are not cast on stone and non-evolutionary but they provide an overview of the functional recovery rate in the different methods discussed here. Indeed, when functional rehabilitation was introduced among patients immobilized for 21 days, both early mobilized groups recovered more or less completely with pain that completely disappeared, without instability nor recurrence. The self-rehabilitation business undertaken in these two groups allowed the avoidance of long and expensive physiotherapist-assisted rehabilitation sessions.

However, these promising results and therapies may be offset by the omnipresence in Senegal of traditional healers who usually do tend to apply controversial if not dangerous self- massages. That is why we have consistently advised our patients to avoid such dubious practices. Indeed, many healer’s massaging methods are responsible for the rising tide of periarticular ossification among patients [20, 23]. Such types of ossification were not found in the series we observed in spite of indomethacin prescription complete ban.

We did not articulate elbow orthotics. Whether for protection or recovery of amplitude of motion, they remained a future step in elbow dislocation management.

There are limitations to our current study. First, its sample size was relatively small due to exclusion criteria eliminating types 2 and 3 fractures of the radial head and of the coronoid process. However, the follow up rate was 100%. In addition, regarding cases with an associated fracture of the radial head or coronoid process type 1, we decided to randomize them in Groups 1 and 2. This choice limited the randomization and balanced distribution processes.

## CONCLUSION

The treatment of closed and stable elbow dislocation remains orthopedic. The risk of instability during the acute phase warrants a 10-day short systematic immobilization followed by early mobilization even in the case of dislocation associated with fractured radial head type 1 of Masson or coronoid process type 1 of Morrey. The prescription for active functional rehabilitation justifies the absence of indomethacin prescription.

## Figures and Tables

**Table 1:** Repartition of groups according to type of contention.

G	Number of patients	Type of contention	Mobilization	Protocol of reabilitation	Evaluations
**1**	21	Splint plast whithin 21 D	From D21	Active motions helped by physiotherapist	D21-30-90
**2**	20	Splint plast whithin 10 D	From D10	Self-reabilitation by active motions and after, helped by physiotherapist	D10-21-30-90
**3**	19	Scarf	From D1	Self-reabilitation by active motions	D7-14-30-90

G= group D= Day

**Table 2:** Functional evaluation results on day 30.

***Groups***	***Number of Cases***	***SCORE OF THE MAYO CLINIC ELBOW PERFORMANCE***				***Total***
		*Excellent*	*Goog*	*Medium*	*Bad*	
***G1***	21 (35%)	0%	4(19%)	7 (33,4%)	10 (47,6%)	*100%*
***G2***	20 (33,3%)	14 (70%)	4(20%)	2 (10%)	0%	*100%*
***G3***	19 (31,7%)	17 (89.4%)	1(5.3%)	1 (5.3%)	0%	*100%*
***Total***	*60 (100*%)	*31 ****(51.6%)***	*9 ****(15%)***	*10 ****(16.7%)***	*10 ****(16.7%)***	*100%*

G1: first group G2: second group G3: third group

**Table 3:** Functional evaluation results on Day 90.

***GROUPS***	***NUMBER OF CASES***	***MAYO CLINIC ELBOW PERFORMANCE SCORE***				***TOTAL***
		*EXCELLENT*	*GOOD*	*MEDIUM*	*BAD*	
G1	21 (35%)	19 **(90.47%)**	2(9.53%)	0%	0%	*100%*
G2	20 (33,3%)	20 **(100%)**	0%	0%	0%	*100%*
G3	19 (31,7%)	19 **(100%)**	0%	0%	0%	*100%*
***Total***	*60 (100*%)	58 **(96.67%)**	2 **(3.3%)**	0%	0%	*100%*

G1: first Group G2: second Group G3: third Group
